# Effect of Surface Treatment Protocols on Nanoindentation-Derived Mechanical Properties and Wettability of Hybrid CAD/CAM Materials

**DOI:** 10.3390/dj14100650

**Published:** 2026-10-08

**Authors:** Miloš Todorović, Bojan Petrović, Stefan Vulović, Nastasija Malivuk, Sanja Kojić, Milan Simić, Dejan Marković

**Affiliations:** 1 Department of Preventive and Pediatric Dentistry, School of Dental Medicine, University of Belgrade, 11000 Belgrade, Serbia; dejan.markovic@stomf.bg.ac.rs; 2 Department of Preventive and Pediatric Dentistry, Dentistry Clinic of Vojvodina, Faculty of Medicine, Department of Dentistry, University of Novi Sad, 21000 Novi Sad, Serbia; 3 Department of Prosthodontics, School of Dental Medicine, University of Belgrade, 11000 Belgrade, Serbia; stefan.vulovic@stomf.bg.ac.rs; 4 Faculty of Technical Sciences, University of Novi Sad, 21000 Novi Sad, Serbia; malivuk.nastasija@uns.ac.rs (N.M.);; 5 Faculty of Sciences, University of Novi Sad, 21000 Novi Sad, Serbia

**Keywords:** CAD/CAM, hybrid materials, polymer-infiltrated ceramic network, surface treatment, nanoindentation, contact angle, wettability, adhesive cementation

## Abstract

**Background/Objectives:** Hybrid CAD/CAM materials combine ceramic and polymeric phases, so conditioning protocols developed for glass–ceramics may not transfer to them. Because wettability and near-surface mechanics are usually studied separately, this study measured both to test whether conditioning trades one for the other. **Methods:** Edelweiss C-Block, Lava Ultimate, Vita Enamic and Cerasmart 270 specimens were left untreated or treated with 9% hydrofluoric acid (HF) for 60 s, sandblasting (50 µm Al_2_O_3_, 1 bar) or silane. Static water contact angle (sessile drop; 90 readings per group) and hardness, elastic modulus and maximum penetration depth (nanoindentation at 30 mN, Oliver–Pharr; 17–25 valid indentations per group) were compared with controls using Welch’s *t*-tests with Holm correction. **Results:** Control contact angles (45.1–60.4°) fell to 6.3–18.1° after sandblasting and 8.8–32.5° after silanization; HF lowered them to 17.9–38.0° in three materials but raised Lava Ultimate’s from 48.4° to 58.1°. Sandblasting caused the largest mechanical losses in every material (hardness reduced by >90%, penetration depth up to 7.9-fold); HF reduced hardness by 58–98%. Silanization preserved 93–97% of control hardness in Lava Ultimate and Cerasmart 270 and gave Vita Enamic its lowest contact angle. **Conclusions:** Improved wettability and preserved near-surface mechanics were not mutually exclusive, and responses depended on microstructure. Bond strength was not measured, so these findings should guide, not replace, bonding studies.

## 1. Introduction

Computer-aided design/computer-aided manufacturing has widened the range of materials available for indirect restorations [1,2]. Beyond monolithic ceramics and polymers, a class of hybrid materials now combines features of both. These include polymer-infiltrated ceramic networks (PICNs) and highly filled nanoceramic composites [3,4]. They were developed to reduce the brittleness of glass–ceramics while improving on the wear resistance of conventional resin composites [3,4]. Their elastic modulus lies closer to that of dentin. They mill and polish readily and can be repaired intraorally. They are now used widely for inlays, onlays, veneers, solo crowns, endocrowns and overlays/table-tops, all chairside and in the laboratory [5,6]. Their mechanical behavior falls between conventional composites and glass–ceramics, which gives adequate fatigue resistance for minimally invasive adhesive restorations [1,2]. Esthetic performance is satisfactory, although long-term clinical data remain limited for the newer products [3,4].

Clinical success depends heavily on the adhesive interface [7,8]. Homogeneous glass–ceramics bond predictably: hydrofluoric acid (HF) etching creates micromechanical retention, and silane provides chemical coupling [9,10]. Hybrid materials are more complex. Ceramic and polymeric phases coexist as interpenetrating networks, or as fillers dispersed within a resin matrix [11,12]. Protocols optimized for feldspathic or lithium disilicate ceramics may therefore not transfer [11,12]. Etching efficacy depends on the volume fraction, distribution and chemistry of the glass phase. The resin component is largely unaffected by HF but may respond to air-particle abrasion or to resin-compatible silane chemistries [13,14]. Material composition and surface treatment interact, and different hybrid structures require different conditioning [5,6].

Among available approaches, HF etching remains the most widely used for ceramic-based CAD/CAM materials, dissolving the glassy matrix to create micro-retentive topography [15]. Silanization then promotes chemical bonding through siloxane formation and improved wetting [16,17]. Non-thermal plasma has also been proposed as a means of raising surface energy and removing organic contaminants without mechanical roughening [18,19]. Plasma activation was not included in the present study, because it is not yet part of routine chairside conditioning and its effect depends on device-specific parameters (working gas, power, distance and exposure time) that would require separate optimization for each material; it is being investigated in a dedicated follow-up study. How these protocols interact with the bi-phasic microstructure of hybrid blocks remains insufficiently characterized [20].

Wettability, measured as static water contact angle, is an indirect indicator of surface energy and of the potential for intimate adhesive contact [21,22]. A lower contact angle after treatment is usually read as favorable, since it reflects better spreading and infiltration of resin cement into surface irregularities [23]. Wettability alone is not sufficient, however. Treatments that raise surface energy may simultaneously damage the near-surface region through microdefects, phase-selective dissolution or alteration of the polymer network [24,25]. Such damage could predispose restorations to subcritical crack propagation under function. Nanoindentation resolves these changes at the scale of the adhesive interface, which conventional bulk mechanical testing cannot reach [26,27].

The literature addressing these two dimensions together remains fragmented. Most studies report either wettability and bond strength [28,29] or mechanical change in isolation [30]. Few combine them within one standardized framework across several hybrid classes, so it remains unclear whether the wettability gained by a given protocol is necessarily paid for by damage to the surface layer in which the adhesive interface will form. Commercial hybrid blocks also vary widely, from ceramic-network systems to resin-dominant nanocomposites, so identical treatments may produce very different responses [31,32]. Material-specific pretreatment is therefore likely to be necessary for predictable bonding and long-term survival [33]. The aim of this study was to evaluate how HF etching, sandblasting and silanization affect the surface wettability and the near-surface mechanical properties of four hybrid CAD/CAM materials with distinct microstructures. The novelty of this study lies in measuring both outcomes for the same four materials under identical conditioning, so that the wettability gained by each protocol can be set directly against its mechanical cost, and in using indentation depth to estimate the thickness of the treatment-affected layer. The null hypothesis of this research is that there is no significant difference in contact angle, hardness, elastic modulus or maximum penetration depth between untreated controls and treated specimens for each material. Contact angle was measured by droplet shape analysis. Hardness and elastic modulus were determined by nanoindentation, based on the recorded load–displacement behavior.

## 2. Materials and Methods

### 2.1. Study Design and Materials

This in vitro study evaluated three clinically relevant surface-treatment protocols on four commercial hybrid CAD/CAM restorative materials (Table 1).

These materials were selected because they represent different hybrid concepts, including polymer-infiltrated ceramic networks and resin-based nanoceramic blocks. For each material, specimens were allocated to four conditions: untreated control, hydrofluoric acid etching, sandblasting and silanization. This gave 16 material–treatment combinations. Two complementary methods were used. Droplet shape analysis provided the static water contact angle as an indicator of wettability, with droplet volume recorded for quality control. Instrumented nanoindentation provided load–displacement behavior, from which hardness and elastic modulus were determined. Untreated specimens served as the baseline for each material. All measurements were made under controlled laboratory conditions, using the same testing sequence, instrument settings and analytical procedures for every group.

### 2.2. Specimen Preparation

Flat specimens of approximately 10 mm × 10 mm and 2 mm thickness were sectioned from the commercial blocks using a Buehler IsoMet 4000 Linear Precision (Lake Bluff, IL, USA.) saw with IsoMet Diamond Wafering Blade 15LC Diamond and 10LC Diamond (according to the manufacturer’s instructions) under continuous water cooling, to limit heat generation and avoid altering the microstructure. Test surfaces were finished and polished with 600-, 800-, 1000- and 1200-grit silicon carbide paper under continuous water irrigation. Polishing followed a standardized figure-of-eight motion for an equal duration on every specimen. Surfaces were then rinsed with distilled water to remove residual abrasive particles. Specimens were ultrasonically cleaned in distilled water for 10 min, rinsed and dried with oil-free compressed air. Each was inspected visually, and specimens with visible defects, contamination, cracks or edge damage were excluded and replaced. In total, 64 specimens were prepared per material (4 per material–treatment group). The remaining specimens were randomly assigned to the four treatment groups and stored under identical conditions. Contact-angle measurements and nanoindentation were performed on separate predefined areas, or on separate specimens where required, so that neither measurement influenced the other.

### 2.3. Surface Treatment Protocols

Control specimens received no treatment beyond standardized polishing and cleaning, and represented the baseline surface condition of each material.

For HF etching, the test surface was treated with 9% HF gel (Ultradent Porcelain Etch) for 60 s. Specimens were then rinsed with water for 60 s to remove acid residues and reaction products, and dried with oil-free compressed air.

For sandblasting, surfaces were abraded with a dental laboratory sandblaster (Hager Werken Airsonic Mini Sandblaster) using aluminum oxide (Al_2_O_3_) particles of 50 µm mean size, at 1 bar from a working distance of 10 mm. This increases surface roughness and removes contaminants, enhancing micromechanical retention [34,35].

For silanization, a silane coupling agent (GC G-Multi PRIMER, GC International, Tokyo, Japan) was applied uniformly according to the manufacturer’s instructions. It was left to react for 20 s, after which excess was gently removed with oil-free compressed air. No adhesive resin was applied before testing.

All three protocols were applied identically to the four materials so that the response of each microstructure to the same challenge could be compared directly; they therefore do not reproduce the manufacturers’ instructions, which differ among these blocks. For Vita Enamic and Cerasmart 270, the manufacturers specify 5% HF for 60 s, whereas HF etching is not recommended for Lava Ultimate or Edelweiss, and the manufacturer of Edelweiss also advises against silanization. The 9% gel, a concentration commonly used for etching silicate ceramics, was chosen as a single standardized challenge and is more aggressive than the protocol recommended for Vita Enamic and Cerasmart 270; this is taken into account in Section 4.6 and Section 4.7.

### 2.4. Droplet Shape Analysis and Contact-Angle Measurement

Wettability was assessed by static contact-angle measurement using the sessile-drop technique, which is widely applied to dental restorative materials. Measurements were made with a KRÜSS DSA25 Drop Shape Analyzer and ADVANCE software v1.14 (KRÜSS GmbH, Hamburg, Germany), at 20 ± 1 °C and constant ambient humidity. Specimens were cleaned with oil-free compressed air immediately before analysis, without altering the treated surface. Distilled water was the probe liquid. Droplets of approximately 2–5 µL were deposited vertically by an automated precision dosing system with a 1 mm capillary. Contact angle was determined by Young–Laplace fitting, which gives reliable profile fitting at this droplet size and minimizes errors from gravitational deformation.

Three independent droplets were deposited on non-overlapping regions of each specimen surface per group. Each droplet was recorded continuously after deposition, and 30 consecutive contact-angle measurements were extracted from the image sequence, giving 90 measurements per experimental group. Group means, SDs and statistical comparisons were based on all 90 readings per group. Because the 30 readings of a droplet are technical replicates rather than independent observations, the droplet is the smallest independent unit of the contact-angle data (Section 4.7). Droplet volume was recorded simultaneously to verify dispensing consistency. All four materials were measured in the untreated control condition and after each treatment, immediately after conditioning and using the same time interval for every specimen.

### 2.5. Nanoindentation Testing

Near-surface mechanical properties were measured by instrumented nanoindentation under dry laboratory conditions at room temperature. A G200 nanoindenter (Agilent Technologies, Santa Clara, CA, USA) fitted with a three-sided pyramidal Berkovich diamond tip (Micro Star Technologies, Huntsville, TX, USA) was used. The instrument was housed in an insulated enclosure to reduce acoustic interference, vibration, air currents and temperature fluctuation.

Specimens were fixed on the sample holder with the treated surface perpendicular to the indentation axis. Indentation sites were selected under the integrated optical microscope, within flat and visually homogeneous regions free of defects, debris, scratches, specimen margins and previous indentations. A motorized x–y stage positioned each region beneath the indenter. Microscope-to-indenter calibration was performed before testing, giving a positioning accuracy of approximately 1.5 µm.

A peak load of 30 mN was applied to all specimens. The loading function comprised loading to maximum force over 15 s, holding at peak load for 1 s and unloading over 15 s. The hold segment stabilized the peak-load response before unloading. Identical parameters were used for all materials and treatment groups, and load–displacement curves were recorded continuously throughout each cycle.

For every material–treatment combination, indentations were programmed on 1 specimen, spaced 100 microns in two axes, sufficiently to prevent overlap of the surrounding plastic-deformation fields [25]. Measurements showing instrumental disturbance, unstable surface contact, excessive curve scattering or interaction with a specimen edge or the underlying support were excluded according to predefined quality criteria. These criteria were defined before testing and applied to each curve individually, before group statistics were calculated. The remaining valid indentations (n = 17–25 per group; Table 2) were used to calculate group means. Each valid indentation was treated as one observation, so n denotes the number of valid indentations per group, not the number of specimens.

Load–displacement curves were analyzed by the Oliver–Pharr method, with emphasis on the unloading segment, which reflects elastic recovery of the tested surface. The initial unloading stiffness S was obtained by fitting the unloading curve with a power-law function. The reduced elastic modulus Er was calculated from S and the projected contact area A:

Er = (S/2β) · √(π/A)(1)

where β is the geometry correction factor for the Berkovich indenter, taken as 1.034. Hardness H was determined as the ratio of the maximum applied load Pmax to the projected contact area:

H = Pmax/A(2)

The elastic modulus of the specimen Es was then derived from the reduced modulus, accounting for the elastic properties of both specimen and indenter:

1/Er = (1 − νs^2^)/Es + (1 − νi^2^)/Ei (3)

where νs and νi are the Poisson ratios of specimen and indenter, respectively, and Ei is the elastic modulus of the diamond indenter. For each group, the primary nanoindentation outcomes were indentation hardness, elastic modulus, maximum penetration depth and the overall loading–unloading response. Individual curves were retained to assess within-group variability, and a representative mean curve was generated for graphical presentation.

### 2.6. Outcome Variables

The primary physicochemical outcome was the static water contact angle (CA, °), determined by droplet shape analysis. Lower values were interpreted as greater wettability by water. Because a single probe liquid was used, surface free energy and its polar and dispersive components were not calculated, and contact angle is reported as a relative measure of wettability only. Mean droplet volume (µL) was recorded as a quality-control parameter. The primary mechanical outcomes were indentation hardness (H, GPa) and elastic modulus (E, GPa), both calculated by the Oliver–Pharr method. Secondary mechanical variables were the maximum indentation depth (nm) and the qualitative characteristics of the load–displacement curves. Qualitative microscopic observations were included as supportive outcomes.

### 2.7. Statistical Analysis

Analyses were performed in Jamovi (Version 2.7.37; The Jamovi Project, Sydney, Australia), which is based on the R statistical computing environment. Descriptive statistics are presented as mean ± standard deviation (SD). Normality was assessed with the Shapiro–Wilk test and homogeneity of variances with Levene’s test. The study followed a factorial design with two independent variables: CAD/CAM material (Edelweiss, Lava Ultimate, Vita Enamic and Cerasmart 270) and surface-treatment protocol (untreated control, hydrofluoric acid etching, sandblasting and silanization). Dependent variables were static contact angle (°), droplet volume (µL), indentation hardness (GPa), elastic modulus (GPa) and maximum indentation depth (nm). Pairwise comparisons between untreated controls and each treatment used Welch’s independent-sample *t*-test, because of unequal sample sizes and heterogeneity of variances. Holm’s correction was applied within each material, and significance was set at *p* < 0.05. Effect sizes are reported as Hedges’ g with 95% confidence intervals (CIs); g was calculated as the difference between the treated and control means divided by the pooled SD, with the small-sample correction, and its CI was obtained from the large-sample approximation of its standard error. Representative load–displacement curves and optical micrographs were evaluated descriptively. Graphs were produced in Jamovi and refined for publication.

No a priori sample-size calculation was performed. The number of valid indentations differed between groups (17–25), because curves meeting the predefined exclusion criteria (Section 2.5) were removed, and Welch’s test was used because it assumes neither equal group sizes nor equal variances. A sensitivity analysis based on the achieved group sizes (two-sided two-sample *t*-test, power 0.80) showed that standardized differences of d = 0.81–0.94 were detectable at α = 0.05 and of d = 0.94–1.10 at the most stringent Holm-adjusted level (α = 0.017). The effects of HF etching and sandblasting exceeded these thresholds, with the exception of hardness in Vita Enamic, whose control group was highly dispersed; the small silane-related changes in Lava Ultimate and Cerasmart 270 (|g| ≤ 0.43, except the elastic modulus of Lava Ultimate) fell below them. Non-significant differences in these comparisons should therefore not be read as equivalence with the control. For contact angle, the readings from one droplet are not independent, so nominal power calculated for 90 readings per group would overstate the effective sensitivity.

## 3. Results

### 3.1. Baseline Mechanical Properties

**Figure 1:**
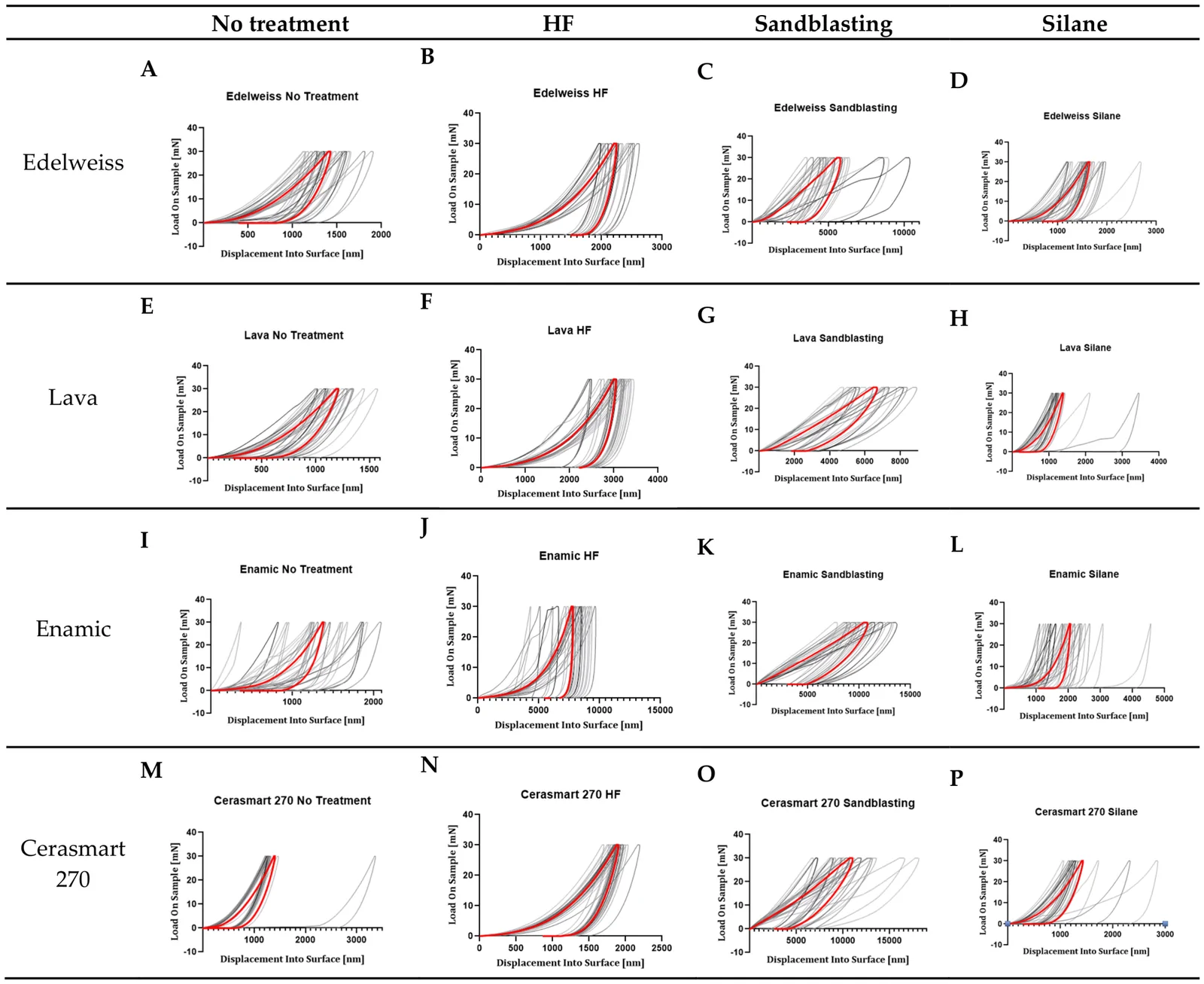
Load–displacement curves of all valid indentations (grey) and the representative mean curve (red) for each material–treatment group, recorded at a peak load of 30 mN. Rows: (**A**–**D**) Edelweiss, (**E**–**H**) Lava Ultimate, (**I**–**L**) Vita Enamic and (**M**–**P**) Cerasmart 270; columns, left to right: untreated control, hydrofluoric acid (HF), sandblasting and silane. Displacement axes are scaled individually for each panel.

Before treatment, the four materials showed distinct mechanical profiles (Table 2 and Figure 1). Vita Enamic recorded the highest mean elastic modulus (34.253 ± 34.794 GPa), but also by far the largest dispersion. Lava Ultimate showed the highest reliably measured hardness (1.163 ± 0.309 GPa), followed by Cerasmart 270 (0.989 ± 0.232 GPa) and Edelweiss (0.797 ± 0.288 GPa); the Vita Enamic mean was nominally higher (1.507 ± 2.959 GPa), but its standard deviation exceeded the mean. Edelweiss showed an intermediate elastic modulus (19.287 ± 3.713 GPa) and Cerasmart 270 the lowest (15.969 ± 2.679 GPa).

**Figure 2:**
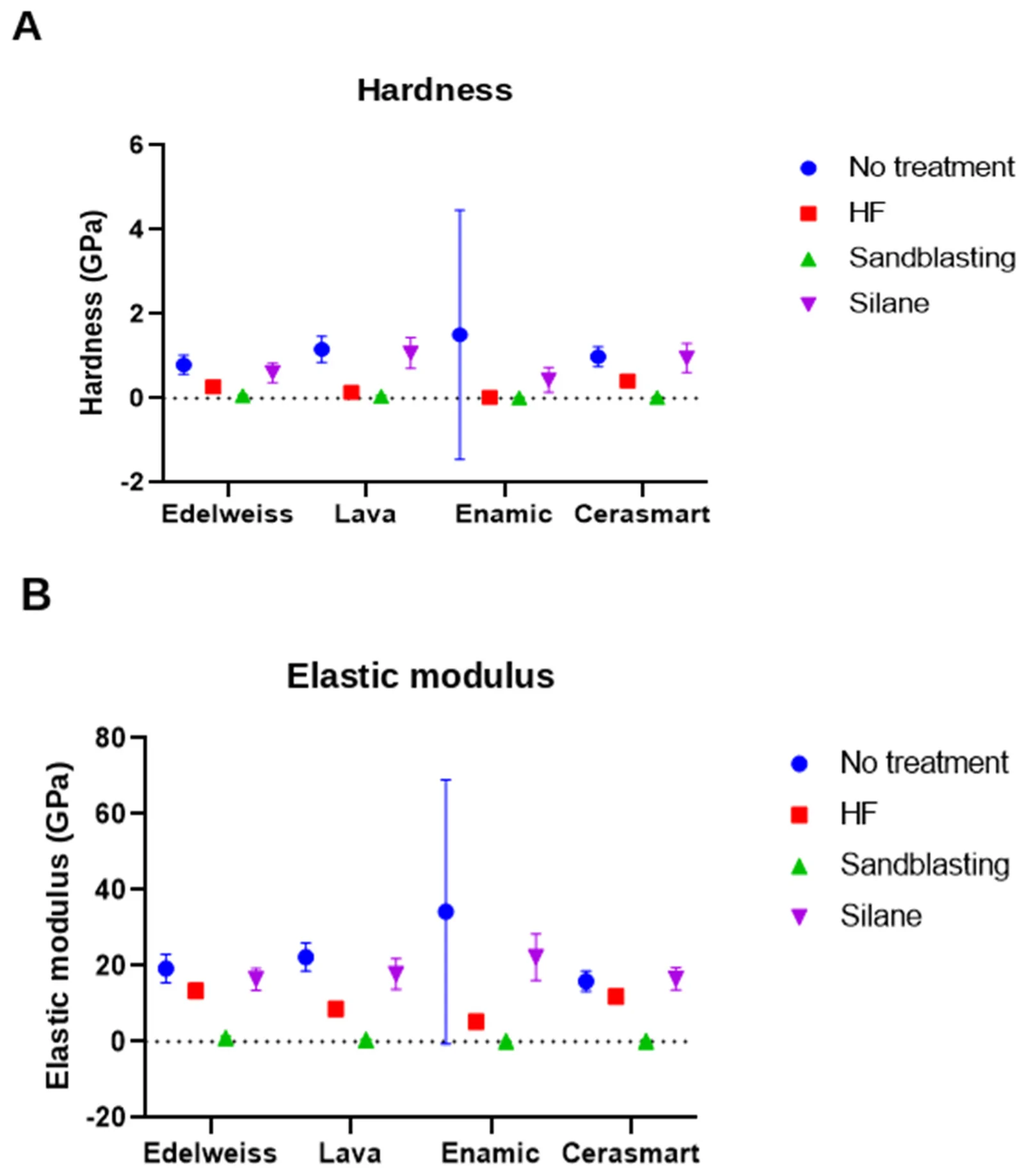
(**A**) Indentation hardness and (**B**) elastic modulus (mean ± SD) of the four hybrid CAD/CAM materials after each surface treatment. The lower error bar of the untreated Vita Enamic group extends below zero because its SD exceeds the mean (Section 3.1).

The untreated Vita Enamic group was exceptionally dispersed. Its standard deviation exceeded the mean for hardness (1.507 ± 2.959 GPa; coefficient of variation ≈196%) and matched it for elastic modulus (34.253 ± 34.794 GPa; ≈102%), whereas the coefficients of variation of the other three untreated materials were 17–36%. The individual curves (Figure 1I) show a broad spread of responses rather than a few outliers, with maximum depths at 30 mN ranging from less than 0.5 µm to about 2 µm. Because the mean ± SD interval extends below zero (Figure 2), the Vita Enamic control mean is not a representative value for this material, and comparisons against this control have correspondingly lower precision: for hardness, none of the differences between the treated groups and this control remained significant after Holm correction, despite reductions of 71–99% in the mean. The origin of this dispersion is discussed in Section 4.3.

Maximum penetration depth reflected these differences. Lava Ultimate penetrated least (1209.2 ± 150.1 nm), indicating the greatest resistance to indenter penetration. Edelweiss (1426.7 ± 200.7 nm), Vita Enamic (1382.7 ± 404.1 nm) and Cerasmart 270 (1401.6 ± 478.3 nm) were comparable, despite differing in hardness and modulus. Because the untreated materials differed intrinsically, all subsequent comparisons were made against the untreated control of the same material.

### 3.2. Effect of Hydrofluoric Acid Treatment

**Figure 3:**
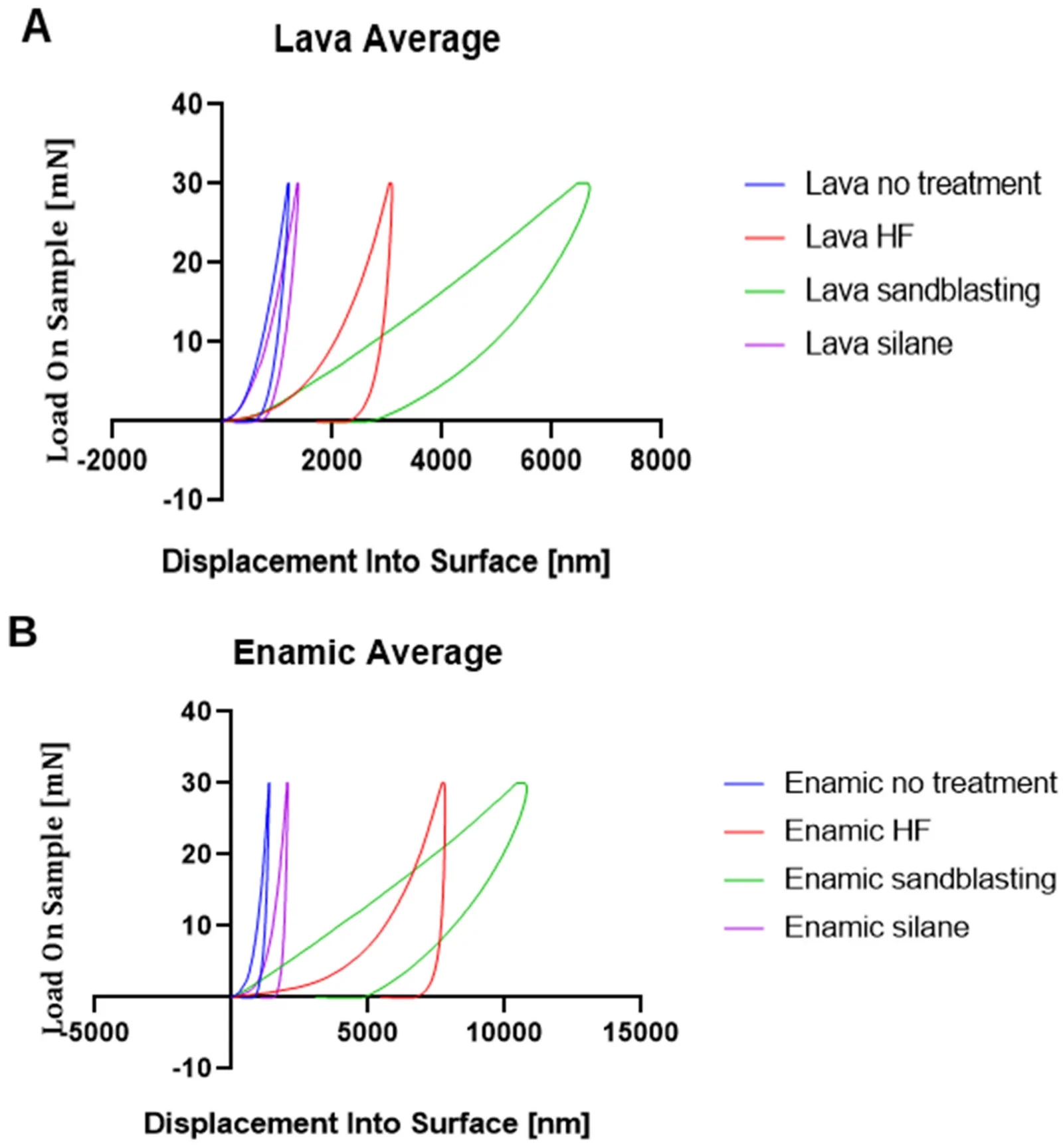

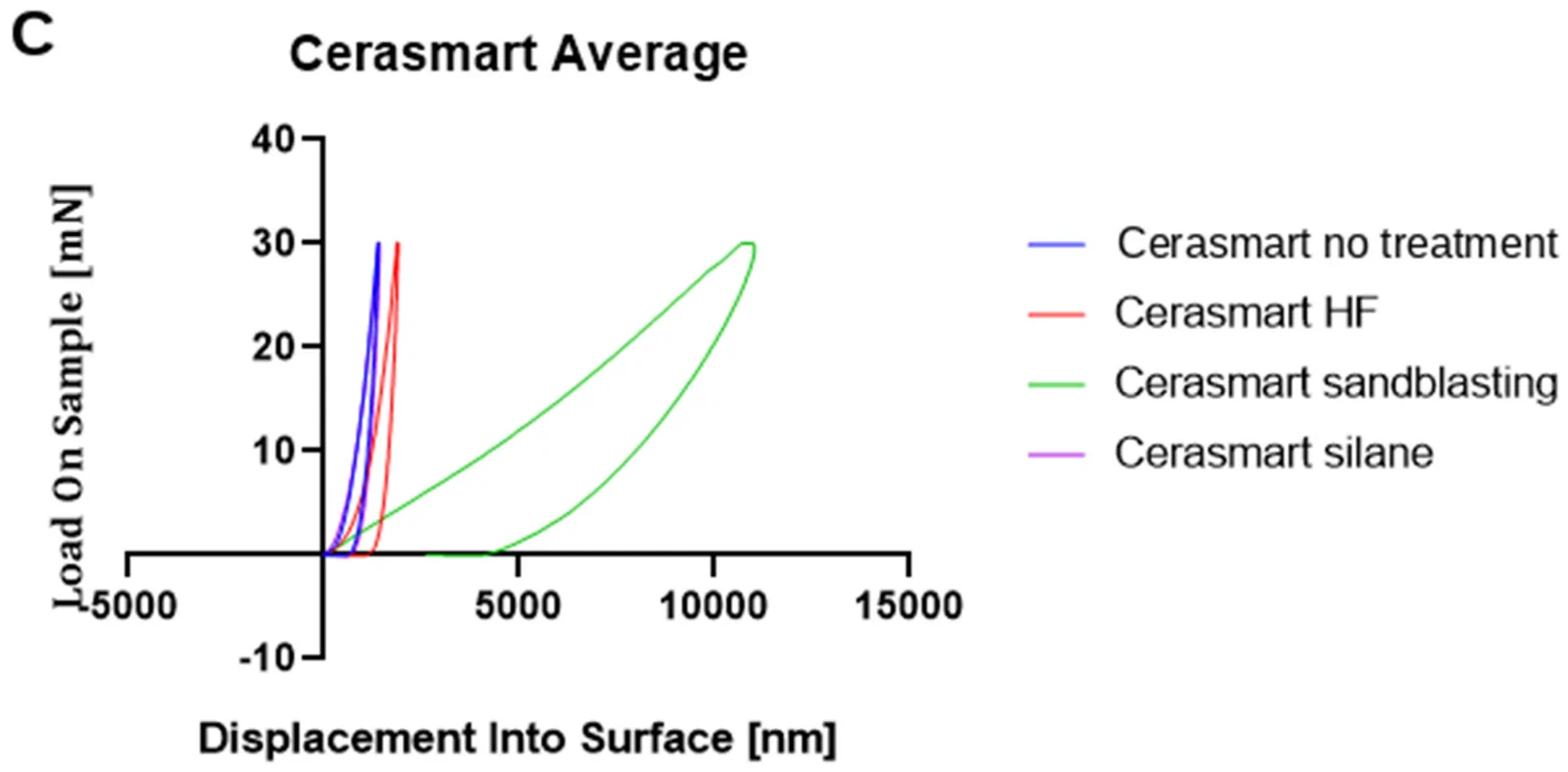
Mean load–displacement curves for each surface treatment at a peak load of 30 mN: (**A**) Lava Ultimate, (**B**) Vita Enamic and (**C**) Cerasmart 270. Displacement axes differ between panels; in (**C**), the silane curve is largely superimposed on the untreated curve. Mean curves for Edelweiss are shown in Figure 1A–D.

Hydrofluoric acid significantly reduced mechanical performance in all four materials (Table 2; Figure 2 and Figure 3). Compared with controls, hardness and elastic modulus fell and maximum penetration depth rose in Edelweiss, Lava Ultimate and Cerasmart 270 (all *p* < 0.001, Welch’s independent-sample *t*-test). Vita Enamic followed the same trend, but the exceptionally high variance of its untreated group weakened the statistical contrast despite the large numerical changes: the fall in elastic modulus and the rise in penetration depth were significant, but the reduction in hardness did not remain significant after Holm correction (Section 3.1).

Vita Enamic deteriorated the most. Elastic modulus fell from 34.253 ± 34.794 GPa to 5.286 ± 1.041 GPa. The hardness decreased from 1.507 ± 2.959 GPa to 0.023 ± 0.013 GPa. Maximum penetration depth rose from 1382.7 ± 404.1 nm to 7804.5 ± 1289.5 nm. Lava Ultimate also lost substantial hardness and elastic modulus, with a 2.6-fold increase in penetration depth (all *p* < 0.001). Edelweiss responded comparably but less severely. Cerasmart 270 retained the highest residual hardness and elastic modulus, indicating greater resistance to acid-induced degradation. Hydrofluoric acid therefore compromised the near-surface mechanical properties of all four materials, but to a material-dependent degree: greatest in Vita Enamic, least in Cerasmart 270.

### 3.3. Effect of Sandblasting Treatment

Sandblasting produced the largest mechanical deterioration in every material (Table 2). Hardness and elastic modulus fell, and maximum indentation depth rose relative to the controls in all four materials; all differences were significant (adjusted *p* < 0.001) except for the hardness of Vita Enamic, which did not remain significant after Holm correction because of the dispersion of its control group (Section 3.1). The direction of the effect was consistent regardless of composition.

Hardness fell from 0.797 ± 0.288 GPa to 0.061 ± 0.032 GPa in Edelweiss, from 1.163 ± 0.309 GPa to 0.049 ± 0.021 GPa in Lava Ultimate, from 1.507 ± 2.959 GPa to 0.017 ± 0.006 GPa in Vita Enamic and from 0.989 ± 0.232 GPa to 0.020 ± 0.010 GPa in Cerasmart 270 (adjusted *p* < 0.001 except Vita Enamic; Section 3.1). Elastic modulus fell to 1.075 ± 0.360 GPa, 0.546 ± 0.161 GPa, 0.207 ± 0.041 GPa and 0.207 ± 0.099 GPa, respectively (all adjusted *p* < 0.001). Maximum indentation depth reached 5780.2 ± 1760.1 nm for Edelweiss, 6694.4 ± 1237.6 nm for Lava Ultimate, 10,818.2 ± 1479.0 nm for Vita Enamic and 11,044.9 ± 2815.7 nm for Cerasmart 270, all significantly greater than the corresponding controls (all adjusted *p* < 0.001).

Sandblasting was significantly more damaging than hydrofluoric acid in every material. The difference was highly significant for Edelweiss, Lava Ultimate and Cerasmart 270 (*p* < 0.001 for all evaluated parameters). Vita Enamic also showed significantly lower hardness after sandblasting (*p* = 0.044), together with markedly lower elastic modulus and greater penetration depth (both *p* < 0.001). Airborne-particle abrasion is therefore a substantially more aggressive conditioning procedure than hydrofluoric acid etching with respect to preserving near-surface mechanical integrity.

### 3.4. Effect of Silanization Treatment

Silanization preserved mechanical properties considerably better than either hydrofluoric acid etching or sandblasting (Table 2). Reductions in hardness and elastic modulus were moderate, and increases in maximum indentation depth were small. The mechanical deterioration after silanization was significantly less than after the other two protocols in all four materials (*p* < 0.05).

Cerasmart 270 and Lava Ultimate remained closest to their untreated controls. Cerasmart 270 recorded a hardness of 0.959 ± 0.343 GPa and an elastic modulus of 16.560 ± 2.951 GPa, with maximum indentation depth virtually unchanged (1430.2 ± 438.7 nm vs. 1401.6 ± 478.3 nm). Lava Ultimate maintained 1.076 ± 0.366 GPa and 17.819 ± 4.062 GPa, with only a limited increase in penetration depth.

Edelweiss also preserved its properties far better than after hydrofluoric acid or sandblasting. Hardness and elastic modulus remained below control values, but both were significantly higher than after the other two procedures (*p* < 0.001), and penetration depth remained markedly lower. Vita Enamic remained the material most affected by conditioning, yet silanization improved every parameter relative to hydrofluoric acid and sandblasting: hardness rose to 0.437 ± 0.297 GPa and elastic modulus to 22.288 ± 6.062 GPa, and maximum indentation depth fell to 2059.1 ± 731.2 nm (all *p* < 0.001).

Across all four materials, silanization resulted in significantly higher hardness and elastic modulus and significantly lower penetration depth than hydrofluoric acid etching or sandblasting. Mechanical preservation followed the order: untreated control > silanization > hydrofluoric acid etching > sandblasting.

### 3.5. Surface Wettability Following Different Treatments

**Figure 4:**
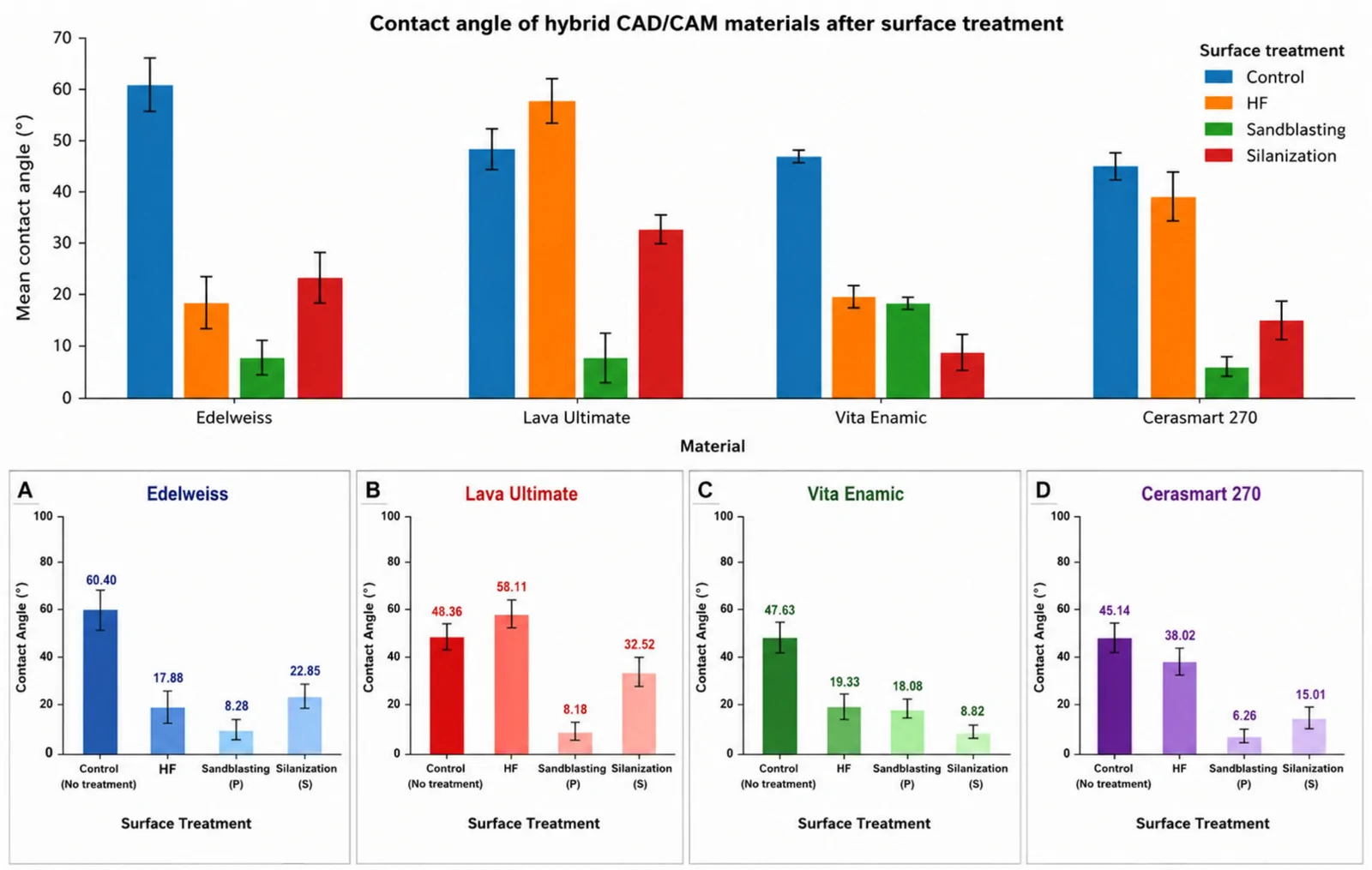
Static water contact angle (mean ± SD) of the four hybrid CAD/CAM materials after each surface treatment, shown for all groups (top) and separately for (**A**) Edelweiss, (**B**) Lava Ultimate, (**C**) Vita Enamic and (**D**) Cerasmart 270.

Surface treatment significantly altered wettability in all four materials (Table 3 and Figure 4). Contact angle changed significantly after every protocol relative to the untreated controls (all adjusted *p* < 0.05). All changes were reductions, with one exception: hydrofluoric acid on Lava Ultimate increased contact angle from 48.36 ± 2.39° to 58.11 ± 2.51°. The magnitude of change varied with both material and protocol.

Sandblasting produced the lowest contact angles in three of the four materials: 8.28 ± 2.20° for Edelweiss, 8.18 ± 3.77° for Lava Ultimate, 18.08 ± 0.32° for Vita Enamic and 6.26 ± 1.04° for Cerasmart 270. These values were significantly lower than after hydrofluoric acid or silanization (all adjusted *p* < 0.05), except in Vita Enamic, in which silanization resulted in the lowest contact angle.

Hydrofluoric acid produced an intermediate and strongly material-dependent effect: 17.88 ± 4.89° for Edelweiss, 58.11 ± 2.51° for Lava Ultimate, 19.33 ± 1.21° for Vita Enamic and 38.02 ± 3.79° for Cerasmart 270. Edelweiss and Vita Enamic were reduced far more than Cerasmart 270, while Lava Ultimate moved in the opposite direction.

Silanization gave 22.85 ± 3.65° for Edelweiss, 32.52 ± 1.19° for Lava Ultimate, 8.82 ± 3.12° for Vita Enamic and 15.01 ± 3.44° for Cerasmart 270. These values were below the untreated controls but remained significantly higher than after sandblasting for most materials (*p* < 0.05). Droplet volumes stayed consistent among groups, confirming standardized dispensing and supporting the reliability of the wettability data (Table 3). Effect sizes (Hedges’ g with 95% CI) for all comparisons against the untreated controls are summarized in Table 4.

### 3.6. Relationship Between Surface Wettability and Mechanical Properties

Changes in wettability were accompanied by changes in near-surface mechanical properties. In general, the protocols that modified the surface most also produced the largest decreases in hardness and elastic modulus and the largest rises in maximum indentation depth. Sandblasting produced the lowest contact angles and the worst mechanical performance simultaneously. Silanization preserved substantially higher mechanical properties at intermediate contact angles, indicating a more favorable balance between surface modification and structural integrity.

Hydrofluoric acid behaved in a material-dependent way. In Edelweiss and Vita Enamic, pronounced reductions in contact angle were accompanied by corresponding mechanical losses. In Lava Ultimate and Cerasmart 270, the change in wettability did not account for the change in mechanical behavior. Increased wettability was therefore generally, but not uniformly, associated with reduced resistance to indentation, and the degree of coupling depended on material composition and on the protocol applied.

## 4. Discussion

### 4.1. Principal Findings

Almost all treatments increased wettability. Sandblasting produced the lowest contact angle in three of the four materials (Lava Ultimate, Cerasmart 270 and Edelweiss) and the largest reductions in hardness and elastic modulus in all four. Hydrofluoric acid and silanization acted in a material-dependent way. Nanoindentation indicated that most protocols modified the near-surface region to depths of 1–11 µm. Whether these changes extend into the bulk of the restoration was not assessed in this study. Conditioning protocols for resin-based CAD/CAM materials are less standardized than those for glass–ceramics. These results provide material-specific evidence to help clinicians balance wettability gains against preservation of surface mechanical properties in a class of restoratives now in wide use.

### 4.2. Surface Wettability After Different Treatments

Published values for untreated hybrid blocks are sparse. One study reports an untreated Lava Ultimate contact angle of approximately 61.7°, and total surface free energies across CAD/CAM materials have been reported from 22.7 mN/m for Vita Enamic to 52.8 mN/m for a zirconia-reinforced lithium silicate [36]. Comparison across studies is limited, because control values, surface-energy components and measurement conditions are reported inconsistently.

Our results agree with parts of the existing literature and conflict with others. Longer hydrofluoric acid etching times progressively lower contact angle in silica-based glass–ceramics, an effect attributed to pore formation and dissolution of the glassy phase [37]. By contrast, sandblasting has been reported to increase water contact angle in resin-based composites, through roughening of an intrinsically hydrophobic resin matrix without a compensating gain in polar character [38]. Our materials behaved differently, which suggests that their glass or ceramic filler content governs the response to mechanical roughening. The rise in contact angle after hydrofluoric acid in Lava Ultimate is consistent with the manufacturer’s advice against acid etching, since its zirconia–silica nanoceramic filler does not etch selectively in the way a continuous glassy matrix does.

Two mechanisms account for the pattern. Sandblasting is purely mechanical. It fractures the surface and preferentially removes resin matrix, exposing inorganic filler of higher intrinsic surface energy. Under the Wenzel model, roughening amplifies whatever wetting tendency a surface already has. In the ceramic-rich materials tested here, exposure of filler outweighed exposure of hydrophobic resin, so contact angle fell. Hydrofluoric acid acts chemically. It dissolves the glassy silica phase, generating microporosity and hydroxyl-rich reactive sites that favor water spreading. This explains its marked effect on Edelweiss, Vita Enamic and Cerasmart 270, all of which contain an accessible glassy or ceramic phase. In Lava Ultimate, the nano-sized zirconia–silica filler is embedded in resin rather than forming a continuous etchable network, so the acid generates little useful porosity and may leave a smoother, more resin-dominated and therefore more hydrophobic surface. Silane acts by chemisorption rather than roughening. Hydrolyzed silane molecules form a thin siloxane network carrying polar silanol (Si–OH) groups, which would be expected to raise surface polarity independently of topography. This explains why silane achieved the lowest contact angle in Vita Enamic without mechanical disruption, and shows that chemical gains in wettability need not involve destructive morphological change.

### 4.3. Nanoindentation Findings

Before conditioning, three of the four blocks separated cleanly on hardness: Lava Ultimate 1.163 ± 0.309 GPa, Cerasmart 270 0.989 ± 0.232 GPa and Edelweiss 0.797 ± 0.288 GPa. Vita Enamic presented a nominally higher mean of 1.507 GPa, but with a standard deviation of 2.959 GPa. Its coefficient of variation was close to 200%, and its modulus behaved the same way (34.253 ± 34.794 GPa). We do not read this dispersion as measurement error. At 30 mN, the maximum penetration depth in untreated specimens was about 1.4 µm, so each indent sampled only a few cubic micrometers. In a polymer-infiltrated ceramic network, the feldspathic skeleton and the infiltrated polymer interpenetrate at exactly this scale. An individual indent therefore lands on a ceramic-rich or a polymer-rich region rather than on a homogenized average [1]. The mean of such a distribution is not a material property. The untreated Enamic values are better read as a broad, probably bimodal population. The other three materials have discrete fillers much smaller than the contact area; their coefficients of variation were 17% to 36%, and they are directly comparable with one another.

Every treatment lowered hardness in every material, but the size of the effect spanned two orders of magnitude. Silanization was close to neutral. Cerasmart 270 retained 97% of its control hardness (0.959 vs. 0.989 GPa) and Lava Ultimate 93% (1.076 vs. 1.163 GPa). In Cerasmart 270, the maximum penetration depth was practically identical to baseline (1430.2 vs. 1401.6 nm). Edelweiss lost about a quarter of its hardness after silane (0.608 GPa), and Enamic fell to 0.437 GPa. Hydrofluoric acid was intermediate and strongly material-dependent. It left 42% of baseline hardness in Cerasmart 270, 35% in Edelweiss, 12% in Lava Ultimate and 1.5% in Enamic. Sandblasting was uniformly destructive. Residual hardness ranged from 7.7% of control in Edelweiss to 1.1% in Enamic. No material retained more than 6% of its baseline elastic modulus: 5.6% for Edelweiss, 2.4% for Lava Ultimate, 1.3% for Cerasmart 270 and 0.6% for Enamic.

The penetration-depth data are the more informative output. They record how far the indenter traveled before the surface could carry 30 mN. In the control and silane groups, that distance was 1.2–2.1 µm. After the addition of hydrofluoric acid, it rose to 1.9 µm in Cerasmart 270 and to 7.8 µm in Enamic. After sandblasting, it reached 5.8 µm in Edelweiss, 6.7 µm in Lava Ultimate, 10.8 µm in Enamic and 11.0 µm in Cerasmart 270. Two consequences follow. The first is interpretive. The treated surfaces were no longer the flat, polished half-space that the Oliver–Pharr formalism assumes [39]. ISO 14577-1 states that surface finish significantly influences instrumented indentation results, and requires a polishing procedure suited to the material [40]. Neither a 9% hydrofluoric-acid-etched ceramic network nor an alumina-abraded composite meets that condition. On a rough, porous or particle-loaded surface, the contact area inferred from displacement exceeds the true load-bearing area, so hardness and modulus are underestimated. An apparent modulus of 0.207 GPa for Vita Enamic cannot be an intrinsic property, since reported values for this material class are 16–28 GPa [1]. It describes compaction of a friable surface layer, not the stiffness of the ceramic network beneath it. We therefore treat all post-treatment values as apparent properties of the conditioned layer.

The second consequence is more useful. The depths set a lower bound on the thickness of the altered zone. The plastic field beneath an indent extends several times the contact depth. An indenter that sinks 11 µm into a sandblasted Cerasmart 270 surface without meeting appreciable resistance is still moving through modified material; it does not reach undisturbed bulk. The converse holds for the silane groups, where depths of 1.4–1.6 µm produced essentially baseline values. A coupling layer a few nanometers thick cannot be resolved at that depth. The small changes recorded after silanization are therefore more plausibly caused by the solvent and monomer fraction of the primer acting on the resin matrix than by the siloxane film itself. The near-identity of silanized and untreated Cerasmart 270 supports this reading.

Were the effects material-dependent? For hydrofluoric acid, unambiguously so. The range of retained hardness, 1.5% to 42%, is wider than any difference among the untreated materials. For sandblasting, far less so. All four converged on a similarly compliant state. Once alumina impact has fragmented the surface, the measured response appears to be governed by the damaged layer rather than by the original microstructure. This agrees with Yoshihara et al. [41], who showed by SEM and STEM that 50 µm alumina exposes and debonds filler and visibly damages composite CAD–CAM blocks, and with Nishigawa et al. [42], who reported material-dependent surface loss after air abrasion. Strasser et al. [36] documented pronounced roughening and surface damage after alumina blasting across ten CAD/CAM materials. Our data add a quantitative estimate of the mechanical cost of that damage. Comparison with published moduli still requires caution, because most values for these blocks come from three-point bending or Vickers indentation and describe bulk rather than near-surface behavior. Coldea et al. [1] obtained 16.4–28.1 GPa and 1.05–2.10 GPa for polymer-infiltrated networks of varying ceramic density, using flexural testing and HV5. Our untreated moduli for Edelweiss (19.287 GPa), Lava Ultimate (22.329 GPa) and Cerasmart 270 (15.969 GPa) fall within that band, which suggests that the baseline measurements were sound.

### 4.4. Relationship Between Wettability and Mechanical Properties

If conditioning worked through a single mechanism, wettability and mechanical integrity would trade off at a fixed rate. Our data match that picture only at the extremes. Sandblasting produced the lowest contact angles in three of four materials and the worst mechanical outcome in all four. The untreated controls sat at the opposite corner. Between those poles, the relationship breaks down, and it does so in three specific ways.

The first concerns the cost of the last few degrees. In Cerasmart 270, silanization reduced contact angle from 45.14° to 15.01°. Hardness fell by 3% (0.989 to 0.959 GPa). Modulus was unchanged within error (15.969 to 16.560 GPa). Penetration depth moved by 2% (1401.6 to 1430.2 nm). Sandblasting reduced the same contact angle to 6.26°. That additional 8.75° cost 98% of the measured hardness and a nearly eightfold increase in penetration depth. The first 30° of wettability gain cost this material almost nothing; the last 9° cost almost everything. Wettability and near-surface damage are not proportional. In Cerasmart 270, the material in which the contrast was cleanest, the marginal mechanical cost of lowering the contact angle rose steeply once treatment moved from chemistry (silane) to abrasion (sandblasting). Because only one HF protocol and one set of sandblasting parameters were tested, whether milder etching or abrasion would shift this threshold remains to be established.

The second is Vita Enamic, for which both outcomes were optimized by the same treatment. Silane produced the lowest contact angle of any protocol in this material (8.82°, against 18.08° after sandblasting and 19.33° after hydrofluoric acid). It also rendered the best-preserved mechanics of any treated Enamic group: 0.437 GPa, 22.288 GPa and 2059.1 nm, against 0.017 GPa, 0.207 GPa and 10,818.2 nm after sandblasting. A single case of this kind is enough to refute an obligatory coupling. It also fits the chemistry. Enamic offers the largest accessible silicate surface of the four materials, so hydrolyzed silane meets the greatest number of surface hydroxyl groups available for condensation, and the gain in wettability costs no material [43]. The silanized Enamic modulus (22.288 ± 6.062 GPa, coefficient of variation 27%) is also a far more plausible figure for this material than the control mean.

The third is Lava Ultimate after hydrofluoric acid, the only group in which a treatment moved both outcomes in the wrong direction. Contact angle rose from 48.36° to 58.11°, leaving the surface more hydrophobic than the polished control. Hardness fell to 12% of baseline, and penetration depth more than doubled. Damage without benefit is difficult to explain unless the two effects have separate origins. Hydrofluoric acid attacks whatever silicate phase it can reach. In a resin nanoceramic, the silica and zirconia nanoparticles are discrete, dispersed and encapsulated within a highly cross-linked matrix rather than assembled into a continuous etchable network. The acid therefore degrades the matrix and the filler–matrix interface without generating the interconnected microporosity that lowers contact angle [44,45]. What remains is a weakened but comparatively resin-dominated, and therefore less hydrophilic, outer surface.

These observations support a simple interpretation. Contact angle is governed by the outermost one to two nanometers of surface chemistry, modulated by topography through the Wenzel relation. Nanoindentation at 30 mN averages the response of one to ten micrometers of material. The two techniques are separated by three orders of magnitude in the depth they probe, and there is no physical reason to expect them to report the same thing. Treatments that act chemically, such as silanization, can change the first without disturbing the second. Treatments that act mechanically necessarily change both. Hydrofluoric acid sits between them, and where it falls depends on whether the material contains a phase for the acid to dissolve. One methodological corollary deserves emphasis. A correlation computed across all sixteen groups would be dominated by the four sandblasted groups, which are extreme in both variables, and would produce a spuriously convincing linear relationship. The decoupling is visible only when the treatments are examined individually.

One caution belongs here. A low contact angle is necessary for a cement to wet and infiltrate a surface, but it is not sufficient for durable adhesion, and bond strength was not measured in this study. A surface that wets perfectly may still fail cohesively within a compliant, friable outer layer, however well the cement is spread across it [32,41]. The mechanical data reported here describe exactly that layer. They complement the wettability results rather than confirm them.

### 4.5. Influence of Material Composition

The responses tracked the microstructure of each block. Vita Enamic, the only true polymer-infiltrated ceramic network in the set, consists of a continuous pre-sintered feldspathic skeleton infiltrated with a dimethacrylate polymer [1,46]; this continuity made it the most etchable material (7.8 µm penetration after HF), explains the scatter of its untreated indentations and offers the largest silicate surface for silane condensation. Lava Ultimate contains discrete silica and zirconia nanoparticles and nanoclusters in a highly cross-linked matrix, with no continuous glass network [44,45]; HF therefore degraded the matrix (88% loss of hardness) without improving wettability, which supports the manufacturer’s advice against etching a physicochemical basis. Cerasmart 270 combines silicate, and therefore silane-reactive, but discrete filler with a resin matrix; this produced the most favorable response to silane and the greatest resistance to HF in the study, but no protection against alumina impact (6.26° and 11.0 µm after sandblasting). Edelweiss, which combines a barium/feldspar glass phase with aluminum and zinc oxide nanoparticles in a polymer matrix, was intermediate on almost every variable, and its high control contact angle (60.40°) suggests a resin-rich polished surface. For this block, the wettability data alone would favor etching, whereas the mechanical data support the manufacturer’s advice against HF; independent characterization of this material remains scarce.

Across the four materials, the best predictor of the response to a given treatment was not total inorganic content but the architecture in which that content is arranged. Continuity of the glass phase determined susceptibility to hydrofluoric acid. Availability of surface silanol groups determined the benefit obtained from silane. Neither predicted the response to air abrasion, which was severe in every case. Treating these products as a single class of hybrid CAD/CAM blocks, and applying one common conditioning protocol to all of them, is not supported by these data [36,47].

### 4.6. Clinical Implications

The question a clinician actually faces is narrow: given this block, on the bench, which conditioning step should precede luting? Three points from the present data bear on it. Because bond strength was not measured, these points are inferences from surface wettability and near-surface mechanical changes alone; they indicate which protocols merit testing in bonding and aging studies, not validated clinical recommendations.

First, contact angle should not be used on its own to select a protocol. Ranked by wettability alone, sandblasting would be the treatment of choice for three of the four materials. Ranked on the combined data, it is the treatment that leaves the surface least able to carry load, and the layer it damages is precisely the layer in which the adhesive interface will sit. The relevant comparison is not which treatment wets best, but which achieves adequate wetting at an acceptable mechanical cost.

Second, the protocol has to follow the microstructure rather than the product category. For Vita Enamic, hydrofluoric acid at the concentration and time used here (9%, 60 s) appeared excessive with respect to preservation of the surface. The manufacturer specifies a lower concentration, and even allowing for that difference, our data indicate that aggressive etching removes a substantial part of the ceramic network on which this material depends. Silane alone resulted in better wettability and far better mechanical preservation, and where an etch is judged necessary, a shorter or more dilute one merits investigation. For Lava Ultimate, the present data support the manufacturer’s advice against hydrofluoric acid and supply a physicochemical rationale for it. For Cerasmart 270, a silane-containing primer alone achieved 15.01° with no detectable mechanical penalty, which is an unusually favorable trade-off; sandblasting improved wettability further, but at a cost that is hard to justify. For Edelweiss, silanization again offered the best balance of the protocols tested, although the manufacturer advises against silanizing this block, so any benefit for bonding requires confirmation.

Third, air abrasion is better regarded as a dose than as a binary step [41,42]. We used 50 µm alumina at 1 bar from a distance of 10 mm, which sits at the conservative end of clinical practice. It still reduced apparent hardness by more than 90% in every material and left altered layers several micrometers deep. Pressure, particle size, working distance and duration all modulate the depth of that layer. Where micromechanical retention is genuinely required, the parameters chosen should be the mildest that achieve it. This is likely to matter most in thin restorations, veneers and minimally invasive onlays in particular, where an altered layer of 5–11 µm represents a larger proportion of the load-bearing thickness.

Two further points are easy to misread from these results. Silanization performed well here on both outcomes, but a primer is a chemical coupling agent and not a substitute for micromechanical preparation; the bond-strength literature consistently shows silane performing best in combination with a conditioning step rather than in place of one [11,43,48]. Moreover, every measurement in this study was made immediately after treatment. The wettability of freshly conditioned surfaces is unlikely to be stable: such surfaces adsorb airborne contaminants, and polymer-containing materials reorganize over time. Whichever protocol is selected, luting should follow it without avoidable delay.

### 4.7. Strengths and Limitations

The strengths of this study lie in its breadth and in the use of two independent quantitative methods applied to the same experimental grid. Four commercial blocks spanning polymer-infiltrated ceramic network, resin nanoceramic and hybrid nanoceramic architectures were tested under four conditions, rendering sixteen material–treatment combinations, with 90 contact-angle readings and 17–25 valid indentations per group, Welch’s tests with Holm correction, reported effect sizes and complementary optical microscopy. The limitations are equally clear. This is an in vitro study on flat, polished specimens. No bond-strength or fatigue testing was performed, so the mechanical measurements cannot be translated directly into adhesive performance. All measurements were taken immediately after conditioning, under dry conditions at room temperature, without water storage, thermocycling or aging; the study therefore says nothing about how the reported differences evolve over time, or about hydrophobic recovery of the treated surfaces. Water was the only probe liquid, which permits contact angle to be reported but not the polar and dispersive components of surface free energy; two or more liquids and an Owens–Wendt or comparable analysis would be required for that. In addition, the 30 contact-angle readings obtained from each droplet are technical replicates, so statistics computed on all 90 readings per group yield narrower confidence intervals than a droplet-level analysis would. Surface roughness was not quantified by profilometry, atomic force microscopy or quantitative image analysis. This is the most consequential omission, because roughness is at once the mechanism invoked in the Wenzel interpretation of the wettability data and the source of the contact-area error that renders the post-treatment indentation values apparent rather than absolute; a roughness dataset would have allowed both to be corrected rather than argued. The mechanistic interpretations offered in this Discussion, namely Wenzel-type amplification of wetting after abrasion and etching and the attribution of reduced apparent hardness and modulus to a damaged surface layer, should therefore be regarded as tentative until confirmed by quantitative roughness measurements. A single peak load of 30 mN was used, so the damaged layer could not be depth-profiled. The very large variance of the untreated Vita Enamic group limits the precision of any comparison against that particular control. The study was not powered a priori, and small effects, such as most of those after silanization, may have gone undetected (Section 2.7). Finally, a single HF protocol (9%, 60 s) was applied to all four materials as a common challenge; it exceeds the concentration specified for Vita Enamic and Cerasmart 270 and departs from the manufacturers’ advice against etching Lava Ultimate and Edelweiss (Section 2.3). Milder, manufacturer-concordant etching, graded sandblasting parameters and non-thermal plasma activation, which was outside the scope of this study (Section 1), remain to be examined.

## 5. Conclusions

Within the limits of this in vitro study, the three conditioning protocols altered the surfaces of four hybrid CAD/CAM materials to very different degrees and at very different mechanical cost. Sandblasting with 50 µm alumina produced the lowest contact angles in Edelweiss, Lava Ultimate and Cerasmart 270 and, in every material tested, the largest reductions in apparent hardness and elastic modulus together with the largest increases in penetration depth. Silanization lowered the contact angle relative to the untreated control in all four materials while retaining 93–97% of the control hardness of Lava Ultimate and Cerasmart 270, and it produced the lowest contact angle of any treatment in Vita Enamic. Hydrofluoric acid acted in a material-dependent manner, improving wettability in the three materials containing an accessible silicate phase but increasing the contact angle of Lava Ultimate, and reducing hardness in all four. Improved wettability and preserved near-surface mechanical properties were therefore not mutually exclusive, and the ranking of the three protocols depended on which of the two outcomes was given priority. Because bond strength and aging were not assessed, these findings indicate which protocols merit evaluation in bonding studies; they do not by themselves establish clinical conditioning protocols.

## Figures and Tables

**Table 1:** Hybrid CAD/CAM materials investigated in this study.

Material	Manufacturer	City, Country	Material Type	Composition
VITA Enamic	VITA Zahnfabrik	Bad Säckingen, Germany	Polymer-infiltrated ceramic network (PICN)	Feldspathic ceramic network (~86 wt%) infiltrated with UDMA/TEGDMA polymer network
Lava Ultimate	3M Oral Care	St. Paul, MN, USA	Resin nanoceramic	Nanoceramic filler particles (~80 wt%) in a highly cross-linked resin matrix
Cerasmart 270	GC Corporation	Tokyo, Japan	Resin nanoceramic	Barium glass and silica nanoparticles (~71 wt%) in a resin matrix
Edelweiss Block-C	Edelweiss Dentistry	Wolfurt, Austria	Direct hybrid composite/CAD–CAM block	Nanohybrid composite material

**Table 2:** Nanoindentation outcomes for the four hybrid CAD/CAM materials by surface-treatment protocol (mean ± SD) and percentage change (Δ) relative to the untreated control of the same material.

Material	Treatment	n	Hardness (GPa)	Δ (%)	Elastic Modulus (GPa)	Δ (%)	Max. Depth (nm)	Δ (%)
Edelweiss	Control	25	0.797 ± 0.288	—	19.287 ± 3.713	—	1426.7 ± 200.7	—
	HF	21	0.276 ± 0.049	−65.4	13.459 ± 1.872	−30.2	2247.5 ± 194.6	+57.5
	Sandblasting	19	0.061 ± 0.032	−92.3	1.075 ± 0.360	−94.4	5780.2 ± 1760.1	+305.1
	Silane	25	0.608 ± 0.231	−23.7	16.433 ± 2.858	−14.8	1645.3 ± 307.2	+15.3
Lava Ultimate	Control	21	1.163 ± 0.309	—	22.329 ± 3.708	—	1209.2 ± 150.1	—
	HF	25	0.145 ± 0.035	−87.5	8.606 ± 1.464	−61.5	3204.3 ± 340.1	+165.0
	Sandblasting	17	0.049 ± 0.021	−95.8	0.546 ± 0.161	−97.6	6694.4 ± 1237.6	+453.6
	Silane	19	1.076 ± 0.366	−7.5	17.819 ± 4.062	−20.2	1382.6 ± 549.8	+14.3
Vita Enamic	Control	21	1.507 ± 2.959	—	34.253 ± 34.794	—	1382.7 ± 404.1	—
	HF	25	0.023 ± 0.013	−98.5	5.286 ± 1.041	−84.6	7804.5 ± 1289.5	+464.4
	Sandblasting	24	0.017 ± 0.006	−98.9	0.207 ± 0.041	−99.4	10,818.2 ± 1479.0	+682.4
	Silane	24	0.437 ± 0.297	−71.0	22.288 ± 6.062	−34.9	2059.1 ± 731.2	+48.9
Cerasmart 270	Control	19	0.989 ± 0.232	—	15.969 ± 2.679	—	1401.6 ± 478.3	—
	HF	25	0.411 ± 0.044	−58.4	11.922 ± 0.769	−25.3	1903.6 ± 95.6	+35.8
	Sandblasting	23	0.020 ± 0.010	−98.0	0.207 ± 0.099	−98.7	11,044.9 ± 2815.7	+688.0
	Silane	19	0.959 ± 0.343	−3.0	16.560 ± 2.951	+3.7	1430.2 ± 438.7	+2.0

n, number of valid indentations per group after exclusion of curves that met the predefined rejection criteria (Section 2.5); these are repeated measurements on 1 specimen per group, not independent specimens. Δ, percentage change relative to the untreated control of the same material. Post-treatment values are apparent properties of the conditioned surface layer (Section 4.3).

**Table 3:** Static water contact angle and droplet volume for the four hybrid CAD/CAM materials by surface-treatment protocol (mean ± SD; 90 readings per group).

Material	Treatment	Contact Angle (°)	Droplet Volume (µL)
Edelweiss	Control	60.40 ± 4.65	6.001 ± 0.277
	HF	17.88 ± 4.89	5.629 ± 2.627
	Sandblasting	8.28 ± 2.20	3.993 ± 1.134
	Silane	22.85 ± 3.65	5.375 ± 2.454
Lava Ultimate	Control	48.36 ± 2.39	3.314 ± 0.998
	HF	58.11 ± 2.51	4.066 ± 0.367
	Sandblasting	8.18 ± 3.77	1.491 ± 0.949
	Silane	32.52 ± 1.19	4.097 ± 0.047
Vita Enamic	Control	47.63 ± 0.44	7.768 ± 0.319
	HF	19.33 ± 1.21	5.661 ± 0.907
	Sandblasting	18.08 ± 0.32	7.505 ± 0.163
	Silane	8.82 ± 3.12	7.150 ± 1.927
Cerasmart 270	Control	45.14 ± 2.05	6.194 ± 0.235
	HF	38.02 ± 3.79	4.944 ± 0.618
	Sandblasting	6.26 ± 1.04	4.548 ± 1.316
	Silane	15.01 ± 3.44	7.305 ± 1.296

Each group comprises three droplets × 30 consecutive readings; readings from the same droplet are technical replicates. Droplet volume was recorded as a quality-control parameter.

**Table 4:** Effect sizes (Hedges’ g with 95% confidence interval) for each surface treatment compared with the untreated control of the same material.

Material	Treatment	Contact Angle	Hardness	Elastic Modulus	Max. Depth
Edelweiss	HF	−8.87 [−9.84, −7.91]	−2.38 [−3.14, −1.62]	−1.90 [−2.60, −1.20]	4.08 [3.06, 5.09]
	Sandblasting	−14.27 [−15.77, −12.77]	−3.30 [−4.22, −2.39]	−6.35 [−7.80, −4.90]	3.68 [2.71, 4.65]
	Silane	−8.95 [−9.91, −7.98]	−0.71 [−1.28, −0.14]	−0.85 [−1.43, −0.27]	0.83 [0.25, 1.41]
Lava Ultimate	HF	3.96 [3.46, 4.46]	−4.77 [−5.90, −3.63]	−4.95 [−6.12, −3.79]	7.24 [5.65, 8.83]
	Sandblasting	−12.68 [−14.02, −11.33]	−4.73 [−5.97, −3.49]	−7.71 [−9.56, −5.86]	6.45 [4.86, 8.03]
	Silane	−8.35 [−9.27, −7.44]	−0.25 [−0.88, 0.37]	−1.14 [−1.81, −0.47]	0.43 [−0.20, 1.06]
Vita Enamic	HF	−30.95 [−34.16, −27.74]	−0.73 [−1.33, −0.13]	−1.21 [−1.84, −0.58]	6.37 [4.95, 7.80]
	Sandblasting	−76.49 [−84.39, −68.58]	−0.73 [−1.33, −0.12]	−1.41 [−2.06, −0.76]	8.30 [6.49, 10.12]
	Silane	−17.35 [−19.16, −15.53]	−0.52 [−1.11, 0.08]	−0.49 [−1.08, 0.11]	1.10 [0.48, 1.73]
Cerasmart 270	HF	−2.33 [−2.71, −1.95]	−3.65 [−4.62, −2.68]	−2.15 [−2.90, −1.40]	1.53 [0.86, 2.21]
	Sandblasting	−23.82 [−26.30, −21.34]	−6.10 [−7.54, −4.66]	−8.60 [−10.53, −6.66]	4.48 [3.34, 5.61]
	Silane	−10.60 [−11.73, −9.46]	−0.10 [−0.74, 0.54]	0.21 [−0.43, 0.84]	0.06 [−0.58, 0.70]

g = (mean of treated group − mean of control)/pooled SD, with small-sample correction; negative values indicate a decrease relative to the control. CIs were obtained from the large-sample approximation of the standard error. Nanoindentation: n = valid indentations per group (Table 2). Contact angle: n = 90 readings per group; because readings from the same droplet are not independent, these intervals are narrower than those that using droplets as the unit of analysis would produce. The very large |g| values for contact angle in Vita Enamic reflect the small within-group SDs of this material (Table 3).

## Data Availability

The data presented in this study are available on request from the corresponding author.
